# Biosynthesis, structure and biological function of cholesterol glucoside in *Helicobacter pylori*: A review

**DOI:** 10.1097/MD.0000000000034911

**Published:** 2023-09-08

**Authors:** Lanchi Zhang, Jingri Xie

**Affiliations:** a Heilongjiang University of Chinese Medicine, Harbin, Heilongjiang Province, China; b The First Affiliated Hospital of Heilongjiang University of Chinese Medicine, Harbin, Heilongjiang Province, China.

**Keywords:** cholesterol-α-D-glucopyranoside, *Helicobacter pylori (H pylori*), Infection

## Abstract

*Helicobacter pylori (H pylori*) is a common pathogen, and about 50% of the world population have been infected with it, so the infection of *H pylori* has been an urgent public health problem worldwide. *H pylori* has evolved a variety of strategies to help itself colonize, adapt to the environment and proliferate. Cholesterol glucoside (CG), a characteristic substance in *H pylori*, is related to the membrane stability, morphology, inflammation induction and immune evasion of *H pylori*. Therefore, CG may be a new target to weaken the infection effect of *H pylori*. The biosynthesis process, structure and biological function of CG specific to *H pylori,* as well as anti-CG drugs are discussed and analyzed in this review, in order to explore whether the inhibition of CG synthesis can be an effective strategy to eradicate *H pylori*.

## 1. Introduction

The infection rate of *Helicobacter pylori (H pylori*), a gram-negative spiral bacterium, is about 50% in the world, but it varies in different countries and races, which is mainly related to personal conditions, health resources, strain characteristics and host diversity.^[1]^ *H pylori* can colonize in the host for a long time, causing chronic infection, acute and chronic gastritis, gastric ulcer, duodenal ulcer or gastric mucosa-associated lymphoid tissue lymphoma. The recognized method to eradicate *H pylori* is triple therapy, that is, 2 kinds of antibacterial agents + proton pump inhibitor. The common used antibacterial agents include amoxicillin, clarithromycin, metronidazole, levofloxacin, etc, and they can destroy the cell wall or inhibit protein synthesis and DNA replication to cause the cell lysis and death, while proton pump inhibitor can maintain the activity of antibacterial agents in the acidic environment in stomach.

However, due to the abuse of antibacterial agents in recent years, especially metronidazole widely used in respiratory infectious diseases, there are more and more drug-resistant strains of *H pylori*, and the eradication rate of triple therapy has dropped from 90% to about 70%.^[2–4]^ According to Maastricht Consensus,^[5]^ the eradication rate below 80% is not recognized. Maastricht Consensus focuses on recommending the tetracycline + metronidazole tetrad regimen as the first-line therapy, and this regimen takes into account the increase in the resistance of *H pylori* to some drugs including levofloxacin and clarithromycin in clinic, as well as the absence of drug resistance testing in this regimen. Recent studies have found a correlation between intestinal flora and *H pylori* infection, so the effect of probiotics as a supplement to eradicate *H pylori* has gradually attracted people attention.^[6]^ The high infection rate, high drug resistance and low eradication rate of *H pylori* mean that there is an urgent need to look for some new antibacterial targets.

Sterol glycoside is a membrane lipid that can be synthesized in plants, fungi, *Myxomycetes* and some animals. Cholesteryl glucoside (CG) is a glycosylated derivative of hydroxylated cholesterol, and like other sterol glycosides, CG is very common in plants, but very rare in mammals and bacteria. There are special CG in *H pylori*,^[7]^ such as cholesterol-α-D-glucopyranoside (αCG), cholesteryl-6’-O-tetradecanoyl-α-D-glucopyranoside (αCAG) and cholesteryl-6’-O-phosphatidyl-α-D-glucopyranoside (αCPG), and αCG can be regarded as the precursors of αCAG and αCPG. The special CG in *H pylori* is involved in the process of maintaining the activity, evading immune system clearance and infecting the host of bacteria. In this review, the biosynthetic process, structure and function of CG, as well as the drugs that inhibit the synthesis of CG are summarized, which may provide ideas for finding new anti-*H pylori* targets and a new way for developing new drugs to eradicate *H pylori*.

## 2. Biosynthesis and structure of CG

### 2.1. Biosynthesis of αCG

Cholesterol is the common raw material of all CGs. However, *H pylori* itself lacks genes or enzymes for the synthesis of cholesterol. CG can be detected in other cells that cannot produce cholesterol, such as human fibroblasts,^[8]^ and sterol glycoside transferases that can catalyze the synthesis of sterol glycoside have also been found in some plants and fungi,^[9,10]^ so the same mechanism of cholesterol utilization may also exist in *H pylori*. At first, it was observed that *H pylori* could be attracted by a high cholesterol and could absorb cholesterol without changing the hydrophobicity of cells.^[11–13]^ Until 2006, Lebrun et al^[14]^ observed that the sequence of HP0421 gene in *H pylori* was similar to that of diacylglycerol-α-glucose glycosyltransferase in gram-positive bacteria, and this enzyme could transfer glucose to acyl group so its function was very similar to that of the synthesized CG enzyme predicted.^[15]^ Therefore, they predicted HP0421 as the gene encoding cholesterol-α-glucosyltransferase (CGT). After HP0421 gene was knocked out, no CG was detected in the culture medium, and the analysis on the centrifuged non-cell homogenate showed that CGT was active in the bacterial lysate and membrane components, but almost undetectable in cytoplasmic components, indicating that CGT should be the enzyme that could use cholesterol and be responsible for the synthesis of αCG in *H pylori*, with a membrane dependence. Finally, the localization and quantitative analysis on CGT by Hoshino^[16]^ and Shimomura^[17]^ confirmed that only the CGT existing in the outer membrane has the enzyme activity.

In order to explore the process of this enzymatic reaction, Lee et al^[7]^ added different concentrations of uridine diphosphate glucose (UDPG) into CGT solutions, and added a certain amount of uridine diphosphate or cholesterol as an inhibitor into the solutions at the same time, in which UDP showed a competitive inhibition with UDPG when UDPG was present, while cholesterol showed a mixed inhibition, indicating that UDPG may bind to the active center of the enzyme, while cholesterol may bind to the sites outside the active center of the enzyme. In addition, the localization analysis showed that CGT transferred UDPG to the third carbon atom of cholesterol. UDPG is the raw material for the synthesis of glycogen in the organism^[18,19]^ and mainly exists in the cytoplasm, so that the central enzyme binding site of CGT needs to be transferred to the cytoplasmic side to bind to it. This binding may alter the conformation of CGT, making it easier to bind to cholesterol, suggesting that CGT in *H pylori* should be a membrane-bound and UDPG-dependent cholesterol glycosyltransferase, and its active site is first exposed to the cytoplasmic side of the cell membrane, then the enzyme conformation changes after forming a complex with UDPG, and the enzyme binds to cholesterol to catalyze the transfer of glucose from UDPG to cholesterol to form αCG.

### 2.2. Biosynthesis of αCAG

αCAG is formed by connecting an acyl group to O6’ of glucose of αCG, and the key enzyme to catalyze the synthesis of αCAG is cholesterol-α-D-glucopyranosise acyltransferase (CGAT). Jan et al^[20]^ identified CGAT coding gene, and its characteristics and functions, demonstrating that CGAT is encoded by HP0499 gene, and the active enzyme mainly exists in the outer membrane. Previously, CGAT was considered to be a phospholipase A1 because it could hydrolyze the ester bond of phospholipids to generate fatty acids.^[21]^ Later, it was found that CGAT not only had the function of catalyzing phospholipids, but also the function of additionally catalyzing acyl transfer to O6’ of glucose,^[20]^ and the acyl transferred by CGAT derived from phosphatidylethanolamine (PE) in cells.^[22]^ It has been found that the activity of CGAT in host cells mainly exists in autophagosomes, late endosome and lysosomes because these organelles are acidic (pH in autophagosomes is about 5.0, pH in late endosomes is about 5.0, an pH in lysosomes is about 4.5), and CGAT is active in pH 3-9, with the highest activity at pH 4.5.^[23]^ The acidic environment in organelles also makes CGAT highly active, leading to a rapid production of a large amount of αCAG.

The enzyme for the synthesis of αCPG is not clear until now, but what can be determined is that this enzyme is also a phospholipase, because the acyl group of αCPG also derives from PE, the same as αCAG.^[22]^ The CG linked with phosphates like αCGs has not been found in other organisms except *H pylori*, and the content of αCPG in the normal spiral *H pylori* is little, but more in the spherical *H pylori*.^[24]^ This enzyme may be as membrane-dependent as CGAT because it uses the same substrate αCG as CGAT.

### 2.3. Structure of CG

αCG can be regarded as the precursor of αCAG and αCPG. CGT obtains cholesterol and transfers glucose to 3-OH of cholesterol to generate αCG, then an acyl or phosphatidyl group is connected to O6’ of glucose, an α-bond between C1 of glucose and O3 of cholesterol is formed to generate αCAG or αCPG, and different fatty acid chains are connected to the acyl and phosphatidyl groups to lead to the diversity.^[22]^ Therefore, αCAG or αCPG can be roughly divided into 3 main bodies in structure: cholesterol ring, acyl or phosphatidyl group and fatty acid chain.

**Figure 1.**
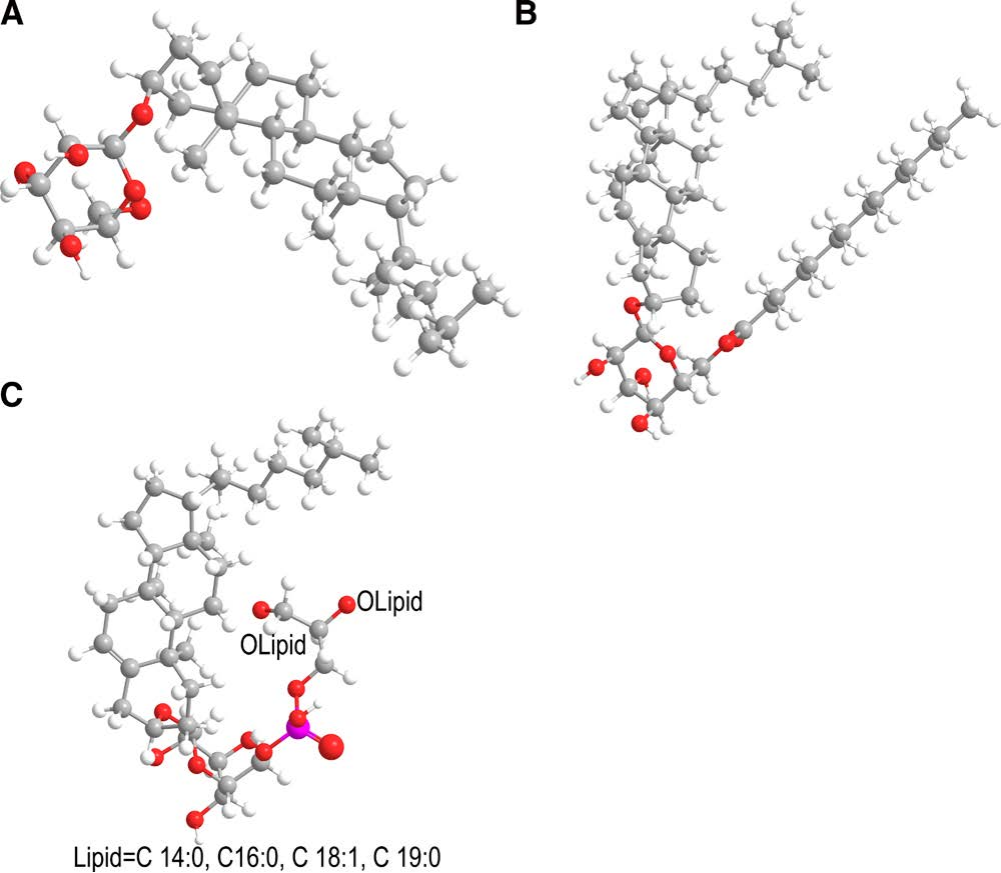
Structure of cholesterol glucoside (CG). (A) cholesterol-α-D-glucopyranoside (αCG), (B) cholesteryl-6’-O-tetradecanoyl-α-D-glucopyranoside (αCAG), (C) cholesteryl-6’-O-phosphatidyl-α-D-glucopyranoside (αCPG), Lipid = C 14:0, C16:0, C 18:1, C 19:0.

αCAG and PE are very similar in that they both exhibit a “hairpin” structure.^[25]^ PE is the main phospholipid component of *H pylori* membrane and playing a key role in the signal transduction and apoptosis of cells.^[26–28]^ The cholesterol ring and acyl group of αCAG are embedded in the bilayer of phospholipids, while the fatty acid chain is located on the surface to facilitate the signal recognition of the membrane. When *H pylori* is cultured alone, C (14:0) is the main fatty acid chain connected to αCAG, and when *H pylori* is co-cultured with host cells, the composition of αCAG will change, with long fatty acid chains, such as 16:0, 18:0 and 18:1. It can be seen that the change in the length of fatty acid chains is caused by the difference in the substrate provided by the host and *H pylori* for the enzyme, and the substrate that forms the short chain comes from *H pylori* itself, while the substrate that forms the long fatty acid chain comes from the host. The structure of αCPG is also “hairpin”-like, and the difference from αCAG is that its cholesterol is connected with the phosphatidyl chain and most of them are connected with fatty acid chains (C14:0, 43.4%) and (C19c:0, 32.5%).^[7]^ Lebrun et al^[14]^ found cholesterol-6’-O-lysophosphatidyl-glucopyranoside (lyso-αCPG) in spherical *H pylori*. The thin layer chromatography showed that the Rf value of lyso-αCPG was smaller than that of the other 3 CGs, and it did not exist in the normal spiral *H pylori* and might come from the αCPG losing 1 fatty acid residue from the perspective of structure.^[24]^ However, the specific biological function of lyso-αCPG is still unknown because the spherical *H pylori* itself is a bacterium with a low biological activity. The structure of CG is shown in Figure 1.

## 3. Biological function

### 3.1. Membrane stabilization

*H pylori* regulates its membrane fluidity and stabilization by relying on the lipid components in the membrane. The phospholipids in its cell wall are mainly PE, and the other phospholipids include cardiolipin, glycerol phosphate and a small amount of phosphatidylserine and sphingomyelin.^[29]^ The cell wall of *H pylori* contains outer membrane phospholipase A (OMPLA),^[30]^ which can be used to form transmembrane channels on the membrane and also catalyze the hydrolysis of PE to produce lysophosphatidylethanolamine. Tannaes et al^[31]^ cultured OMPLA-defected and normal *H pylori* strains, respectively, and found that no matter whether *H pylori* could produce lysophospholipid, αCG and αCPG could be detected, and αCAG could be only detected in strains that could produce a large amount of lysophospholipids. OMPLA is widely found in gram-negative bacteria, but the enzyme is generally in a dormant state. For example, OMPLA in *Pseudomonas aeruginosa* will be assembled and activated only after detecting the attack of other bacteria, while OMPLA in *H pylori* can spontaneously decompose membrane phospholipids into lysophospholipids.^[32]^ Studies have shown that αCPG is the most hemolytic of the 3 CGs.^[7]^ The increase in the content of lysophospholipid will increase the fluidity and permeability of cell membrane^[33]^ and affect the normal morphology of cells,^[34]^ indicating that a high proportion of lysophospholipid is dangerous to cells, so αCG is transformed into αCPG in the environment without lysophospholipid in *H pylori*, then αCPG is utilized to maintain the flexibility of the membrane, and the expression of αCAG in the environment of an increased membrane fluidity can stabilize the cell membrane to prevent its leakage.

### 3.2. Participating in the change of bacterial morphology

Pathogens can be deformed under uncomfortable conditions. For example, *Escherichia coli* may become round in an environment in which it is not easy to survive,^[35]^ while *H pylori* will become spherical in an environment that is not conducive to its survival. Although this “spherical” is very low in activity and cannot secrete urease, the genetic material can be preserved, and when the environment is suitable for survival, it will turn into active to invade the spiral *H pylori* in the host.^[36]^ However, *H pylori* that lacks CGT completely exists in spherical form,^[37]^ indicating that CG can also affect the morphology of bacteria. The growth of bacteria goes through 3 stages: logarithmic stage, steady state stage and declining stage. Shimomura et al^[38]^ cultured *H pylori* under anaerobic conditions to observe the changes of various CGs during the transformation of bacteria from spiral to spherical. They found that in the logarithmic stage, αCPG hardly existed and αCG was transformed into αCAG, in the steady state stage, the increased level of αCPG is higher than that of αCAG, and αCG was mainly transformed into αCPG, in the declining stage, the growth rate of αCPG and αCAG was parallel, and αCG in spherical *H pylori* was transformed into αCAG and αCPG averagely. This deformation process is also accompanied by the change in the phospholipid composition of cell wall. PE is the most abundant lipid in *H pylori*, accounting for about 66% of the total lipids. However, during deformation, the proportion of PE decreases to 29%, and *H pylori* is converted into a “sphere” with low activity rather than bacterial lysis. Because the synthesis of αCPG also requires PE to provide acyl group, the hydrolysates of PE may be used for the synthesis of αCPG in this process, transforming the spiral form into a spherical one.

### 3.3. Formation of lipid raft

Lipid rafts are lipid micro domains rich in cholesterol and sphingomyelin on the outer side of eukaryotic cell membranes, about 70 nm in size and with a certain recognition function, and can mediate some biological processes, such as signal transmission and cell polarization.^[39,40]^ Lipid rafts have been found on the cell membranes of prokaryotes such as *E coli*,^[41]^ *Staphylococcus aureus*,^[42]^ and *Borrelia burgdorferi*.^[43]^ Lipid rafts are the entrance of many pathogens including *H pylori*^[44,45]^ and the “bridge” for these pathogens to invade host cells, and can affect the integrity and morphology of membranes.^[46]^ Huang et al^[29]^ separated all lipids in *H pylori*, including αCG, αCAG, αCPG and cholesterol, and found that αCAG, αCPG and cholesterol all could form an ordered and disordered lipid bilayer structure on the membrane, very similar to the lipid rafts in eukaryotic cells, but there were some differences in the stability of the rafts: αCAG being the strongest and αCPG the weakest. PE could not be utilized to form lipid rafts, and the resistance of fat raft to heat was enhanced after it mixed with αCAG, even stronger than that of domain formed by αCAG alone. The fatty acid chains connected to αCAG and αCPG were different, that is, there were 98.5% fatty acid chains (C14:0) in αCAG, while 43.4% fatty acid chains (C14:0) and 32.5% cyclopropane fatty acids chains (C19:0 cyc) in αCPG. In red blood cells and some viruses, long chain palmitoyl proteins are the key to forming lipid rafts, because palmitoyl groups provide a high energy in the process of separating lipid rafts from cell membranes, which is conducive to the binding of macromolecules to the lipid rafts.^[47]^ The same situation should exist in the formation of lipid rafts, that is, the long-chain fatty acids provide a higher energy, which is more conducive to the segmentation of the bilayer between the rafts and disordered lipids, and the aggregation of related proteins. In addition, the rigid sterol ring of cholesterol is more likely to interact with the long chain, thus affecting the conformation of adjacent hydrocarbon chains to result in the consolidation and lateral reorganization of some membrane structures in order to form lipid rafts.^[48]^ From the perspective of structure, cyclopropane fatty acid (C19:0 cyc) is different from the long chain structure, and it is difficult for its ring group to bind to the ring in cholesterol aglycone, so it is difficult for a high proportion of αCPG to be utilized to form lipid rafts.

### 3.4. Inducing inflammatory reaction and escaping immune clearance

The 2 virulence factors of *H pylori*, CagA and VacA, are the key to pathogenesis, and *H pylori* in which these 2 genes can be expressed seems to be more closely related to the occurrence and development of ulcers.^[49]^ The isolated *H pylori* strains in clinic can be divided into 2 types according to the different expression genes. Both genes are expressed in type I, and neither is expressed in type II.^[50]^ Tannaes et al^[25]^ cultured *H pylori* in a neutral (pH = 7.4) medium, and added hydrochloric acid in the experimental group to adjust the pH to pH = 5, and it was found that different pH could affect the invasion ability of *H pylori*. The bacteria in neutral media exhibit an inability to release VacA and urease and to adhere to or invade epithelial cells, called L-type, while the addition of hydrochloric acid converts almost all L-type *H pylori* into invasive bacteria, called S-type. They studied the lipid components of 2 types of bacterial membranes, and the results showed that αCPG was dominant in the L-type *H pylori* (accounting for 72% of all membrane lipid components), while αCAG was dominant in the S-type *H pylori* (accounting for 65% of all membrane lipid components), suggesting that in addition to genotype, CG will also affect the ability of *H pylori* to infect the host. The *H pylori* with a high αCAG level has a stronger activity and can adhere to the host cells to infect them, and on the contrary, the *H pylori* with a higher αCPG level has no ability to invade the host. CagA is mainly transmitted to host cells by T4SS in *H pylori*.^[51]^ Wang et al^[52]^ reported that in CGT gene-knocked out *H pylori* strain, the CagA translocation, tyrosine phosphorylation and hummingbird phenotype formation could be not induced, nor could be recruited the cholesterol on the host cell membrane to form lipid rafts, and the longer the length of the acyl chain, the more significant the CagA translocation,^[22]^ demonstrating that the fat raft formed by CG is an important component of bacteria to transmit virulence factors and induce inflammation. In addition to CagA and VacA, some signal pathways in the pathogenic process of *H pylori*, such as IL-33/ST-2/IL-1, are also activated through ST-2 recruitment by lipid rafts.^[53]^ When bacteria adhere to the host cell membrane, CGT and CGAT absorb cholesterol to form αCAG, the membrane of the adhesion site is selectively laterally separated and reconstituted to form lipid rafts, and the lipid rafts act as sensitive signals to deliver CagA, VacA and various inflammatory factors to the host cytoplasm, inducing the downstream signal activation and phenotypic changes.

*H pylori* can induce gastric epithelial cells to produce human β-defensin 3, activate defensive MAPK, JAK/STAT signaling pathways and stimulate the secretion of IFN-γ,^[54–56]^ playing the role of clearing *H pylori*. Surprisingly, despite the existence of multiple antibacterial mechanisms, *H pylori* can still cause a persistent infection, and this long-term infection state can also induce gastric cancer.^[57]^ CGT plays an important role in resisting immune system clearance. As mentioned above, CGT can intercept cholesterol on the membrane of host cells and bind it to itself, and this consumption affects the host cholesterol-dependent antibacterial process. For example, subunits (IFNAR1/IFNAR2 and IFNGR1/IFNGR2) of IFN type I (a/b) and II (g) are assembled in lipid rafts,^[58,59]^ and with the depletion of cholesterol, IFN receptor subunit assembly fails, which may reduce the signal transduction of IFN-γ, leading to a decrease in the phosphorylation of JAK and STAT1.^[60]^ Therefore, CG has various effects on the virulence of *H pylori*, such as participating in the formation of “bridge” lipid rafts, mediating the adhesion of bacteria to the host, delivering virulence factors and inducing inflammatory reaction, and escaping the clearance of the host immune system to cause a persistent infection.

### 3.5. Activating C-type lectin

C-Type lectin can interact with various endogenous and exogenous ligands to participate in the immune process of the body.^[61]^ Both Mincle and dendritic cell immunoactivating receptor (DCAR) are C-type lectin receptors, and Mincle specificity is to recognize lipids and glycolipids,^[62]^ while DCAR can recognize phosphatidylinositol mannoside to cause Th1 reaction.^[63]^ Mincle contains a carbohydrate recognition domain CRD^[64,65]^ that can recognize pathogen-related molecular patterns, and a cholesterol recognition amino acid consensus motif.^[63]^ The structural analysis of CRD has revealed the mechanism that Mincle recognized the combination of sugar and lipid. CRD has a typical sugar binding site centered on Ca^2+^, containing an EPN (Glu-Pro-Asn) motif that can bind to a glucose residue, a hydrophobic tank next to it to bind the acyl, and a glucose binding site on the other side. This mode of 2 glucose residue binding sites increases the affinity of sugar, and the affinity of acyl at the hydrophobic tank of CRD depends on the side chain length of the ligand.^[66,67]^ Nagata et al^[24]^ first confirmed that αCAG is an effective ligand of human Mincle, and can stimulate dendritic cells and macrophages to up-regulate the expression of inflammatory factors, such as TNF-α and IL-6,^[68]^ activate natural killer T cells^[69,70]^ and aggravate inflammations. Timmer et al^[71]^ further proved that CRD was the preferred Mincle binding site of αCAG, and αCG could not activate Mincle although it could induce an inflammation. Structurally, αCAG is different from Mincle true ligand trehalose dimycolate, which does not have a cholesterol-like ring structure,^[72]^ so binding of αCAG to trehalose dimycolate may be achieved by binding to the hydrophobic groove of CRD via a long fatty acid chain provided by the host cell.

αCPG, different from αCAG, can activate DCAR instead of Mincle.^[71]^ There are key residues Ala136, Gln198 and an atypical Ca^2+^ binding group EPS (Glu-Pro-Ser) around the ligand binding site of DCAR.^[73]^ The hydrophobic tank extending from the ligand binding site is the main difference between DCAR and Mincle. The hydrophobic tank of DCAR can bind to phosphatidyl, meaning that it can recognize the αCPG containing phosphoglycolipids instead of αCAG.

### 3.6. Autophagy

Autophagy is an adaptation and self-protection reaction of cells, and a process in which cell components are degraded by lysosomes and used by themselves.^[74]^ After bacteria invade host cells, they are first isolated in the double membrane vesicles of autophages and then fuse with lysosomes to form autolysosomes, and the hydrolases in the autolysosomes degrade the contents into small molecules and the cells can use the degradation products to provide substrates for their energy metabolism and synthesis.^[75]^ The degradation of pathogens by autophagosomes contributes to immune defense, playing a protective role in infectious diseases.^[76]^ It was previously believed that *H pylori* was an extracellular bacteria, and it was not until 2005 that researchers found *H pylori* in the gastric epithelial progenitor cells.^[77]^ Intracellular *H pylori* is an important factor that causes a persistent inflammation and its recurrence,^[78]^ so the way bacteria enter the host has become an issue that people are eager to understand. Studies have shown that some viruses can use autophagy for self-protection and proliferation, such as autophagy membrane that participates in the nucleocapsid assembly and the release of hepatitis B virus.^[79]^ The autophagy induced by poliovirus can increase the viral load of fibroblasts.^[80]^ Because of this characteristic, autophagy has become one of the important mechanisms for studying the entry of pathogens into the host.

Lai et al^[81]^ reported that *H pylori* could induce the expression of early endosome antigen 1 and autophagy-related protein 12, and compared with that in CGT gene-knockout strain (ΔCapJ), the expression of lysosome-associated membrane protein 1 decreased and the expression of LC3-II in macrophages increased in wild type strain (WT *H pylori*). LC3-II is a marker of autophagy,^[82]^ and can initiate an autophagy together with autophagy-related proteins SQSTM1/p62 and Beclin-1.^[83,84]^ The above results indicate that *H pylori* containing CGT interferes with the degradation of lysosomes in macrophages, but does not affect the formation of autophagy. In order to clarify the specific components of *H pylori* to enhance autophagy, Muthusamy et al^[23]^ used ΔCapJ strain to infect AGS cells pre-added with αCG, αCAG or αCPG, and detected LC3B-II by Western blot, in which the increase of LC3B-II protein level was considered to indicate an enhanced autophagic reaction. The results showed that the autophagic reaction of AGS cells pretreated with αCAG was higher than that of the other cells. However, just wrapping *H pylori* in autophagosomes cannot completely explain that bacteria use autophagy to proliferate, and normally autophagosomes will fuse with lysosomes to degrade the contents, so other factors are needed to interfere with the autolysosome fusion process to ensure that bacteria will not be degraded, which reminds us of *H. pylori* virulence factor, VacA. VacA, a pore-forming toxin related to the endoplasmic and mitochondrial membranes,^[85]^ can inhibit the lysosomal calcium channel protein TRPML1 to destroy the transport process of autolysosomes, so as to form defective lysosomes in cells.^[86]^ To sum up, in the process of *H pylori* intervention on autolysosomes, αCAG increases the formation of autophagosomes containing *H pylori*, while VacA destroys the elimination of *H pylori* by lysosomes, leading to a long-term survival of bacteria.

## 4. Drugs acting on CG

It can be seen that CG is the key to the survival of *H pylori,* and mainly strengthens the cell wall structure of bacteria to resist the external uncomfortable environment and help bacteria to proliferate. At present, few drugs that act on CG have been reported. It is only found in 1 study that cholesterone produced by human intestinal microorganism *E coli* and *Bacteroides* has an anti-*H pylori* activity, and the oral administration of cholesterone alone has an eradication effect of *H pylori* in mice infected with *H pylori* and cholesterone also has a bactericidal effect on isolated drug-resistant strains in clinic.^[86]^ Cholesterone, similar to cholesterol in structure, can competitively bind to CGT to inhibit the generation of αCG and its derivatives. Cholesterone, different from traditional antibiotics in destroying the structure of bacterial cell wall (such as amoxicillin) to lyse bacteria, mainly affects the normal synthesis of *H pylori* cell wall to make bacteria turn into nontoxic and non-proliferative spherical bacteria. Importantly, cholesterone has almost no toxicological effect, and animal experiments have shown that cholesterone can regulate the lipid metabolism in mice and the mice fed with it are healthy, indicating that it is safe,^[87]^ which is also one of the advantages of cholesterone as a new drug to eradicate *H pylori*. However, the clinical application of cholesterone still needs to be studied on its dosage, administration period, safety and stability in an acidic environment.

## 5. Conclusion and prospect

CG, an important component in the biological membrane of *H pylori*, has become a new field of studying the interaction and signal transduction between bacteria and host cells. CG plays a role in maintaining the morphology, membrane stability, lipid raft formation, inducing inflammation and regulating the host autophagy of *H pylori*, so the study on the synthesis of CG is of great significance for understanding the pathogenicity of *H pylori*. Current research results suggest that αCAG may play a more important role in the infection with *H pylori*, while αCPG is considered to be a sign of low activity because αCPG exists more in spherical *H pylori*. αCAG and αCPG are also important components that regulate the permeability of *H pylori* cell wall, and inhibiting the synthesis of CG can significantly eradicate *H pylori*. At present, the mechanisms of CG involved in the synthesis of the cell wall of *H pylori* have been studied more frequently, while the other effects of CG have been less studied, and few anti-CG drugs have been used in humans. There are 2 directions in the research on the eradication therapy of *H pylori* targeting CG. One is to give a certain amount of exogenous cholesterol to supplement the cholesterol consumption by *H pylori* and the cholesterol needed by immune factors to play the role, and the other is to make *H pylori* suffer from cholesterol starvation to inhibit the synthesis of CG. Therefore, the further exploration of CG may provide a new therapeutic way for anti-*H pylori*.

## Author contributions

**Writing – original draft:** Lanchi Zhang.

**Writing – review & editing:** Jingri Xie.
